# FoxO1-negative cells are cancer stem-like cells in pancreatic ductal adenocarcinoma

**DOI:** 10.1038/srep10081

**Published:** 2015-06-11

**Authors:** Weifeng Song, Qi Li, Lei Wang, Weiyi Huang, Liwei Wang

**Affiliations:** 1 Department of Medical Oncology, Shanghai First People’s Hospital, Shanghai Jiao Tong University, Shanghai 200080, China; 2 Shanghai Key Laboratory of Pancreatic Diseases Research, Shanghai 200080, China

## Abstract

Flow cytometry assays using aldehyde dehydrogenase (ALDH) activity or CD133 positivity to isolate cancer stem cells (CSCs) are widely applied but have limitations. Thus, characterization of CSC makers for a specific cancer is potentially important. We have previously shown that miR-21 regulates cancer cell growth via FoxO1 in pancreatic ductal adenocarcinoma (PDAC). Here, we areported evidence of FoxO1-negative PDAC cells as CSCs in PDAC. Both ALDH-high and CD133-high cell fractions isolated from PDAC of the patients expressed high levels of miR-21 and null FoxO1. Cultured PDAC cells were virally transduced with GFP under FoxO1 promoter. GFP (FoxO1)-null PDAC cells expressed high levels of miR-21, and grew more quickly than FoxO1-positive PDAC cells. Moreover, the fold increases in growth of FoxO1-negative vs FoxO1-positive cells were greater than CD133-high vs CD133-low cells, or ALDH-high vs ALDH-low cells. Further, FoxO1-negative cells formed tumor spheres in culture and developed tumors after serial adoptive transplantation into NOD/SCID mice, while the FoxO1-positive cells did not. Finally, selective elimination of FoxO1-negative cells completely inhibited the growth of PDAC cells. Together, these data suggest that FoxO1-negative cells as CSCs in PDAC, and targeting FoxO1-negative cells in PDAC may provide better therapeutic outcome.

Epithelium-origin carcinoma account for majority of malignant cancer worldwide. Pancreatic ductal adenocarcinoma (PDAC) is a highly malignant carcinoma with an extremely high lethality^1^,^2^,^3^. Moreover, compared to other cancers from digestive system, the therapeutic outcome of PDAC is inferior, largely resulting from our poor understanding of the molecular carcinogenesis of PDAC^1^,^2^,^3^.

Cancer stem cells (CSCs) are cancer cells with characteristics of stem cells. CSCs are tumorigenic, and are responsible for cancer relapse and metastasis^4^,^5^,^6^,^7^. Treatments targeting CSCs are believed to improve the current therapy on rapidly growing cancers and highly metastatic cancers^4^,^5^,^6^,^7^. To date, cell surface markers are generally used for isolation of CSCs by flow cytometry. Among these markers, the best established ones are prominin-1 (CD133) and aldefluor, the latter of which is a feature of increased cellular aldehyde dehydrogenase (ALDH) activity. CD133 is expressed in hematopoietic stem cells, endothelial progenitor cells, neuronal, glial stem cells, and CSCs from various tumors^8^,^9^,^10^,^11^,^12^. However, later studies have reported the difficulties in purifying CSCs by CD133^13^. Increased activity of ALDH1, a detoxifying enzyme responsible for the oxidation of intracellular aldehydes^14^,^15^, has also been used in identification of stem/progenitor cells or CSCs^16^,^17^,^18^,^19^,^20^,^21^. However, recent evidence suggests the presence of aldefluor-positive cells in other populations, e.g. proliferating pancreatic beta cells^22^,^23^. Moreover, both CD133 and ALDH/aldefluor methods are lack of cancer specificity. Thus, neither CD133 nor ALDH/aldefluor is a perfect method for isolation of CSCs.

The microRNAs (miRNAs) consist of about 22 nucleotides, and play a substantial role in the regulation of protein-coding gene expression to affect cell proliferation, apoptosis and differentiation^24^,^25^,^26^. MicroRNA-21 (miR-21) was recently reported as an aberrantly expressed miRNA in PDAC^24^,^25^,^26^, but the precise involvement in the molecular events is not completely understood. Forkhead box protein (Fox) proteins are a subgroup of the Forkhead family of transcription factors with a conserved DNA-binding domain^27^. Among Fox proteins, FoxO1 transcription factor plays a critical role in cell-cycle control^27^. Recently, we reported that miR-21 binds to FoxO1 mRNA on its 3’UTR to prevent its translation. Loss of FoxO1 results in attenuated inhibition on cell growth^28^.

Here, we reported that ALDH-high and CD133-high cell fractions isolated from PDAC of the patients were FoxO1-negative. Cultured PDAC cells were then virally transduced with GFP under FoxO1 promoter. GFP (FoxO1)-null PDAC cells expressed high levels of miR-21, and grew more quickly than FoxO1-positive PDAC cells. Moreover, FoxO1-negative cells formed tumor spheres in culture and developed tumors after serial adoptive transfer into NOD/SCID mice, while the FoxO1-positive cells did not. Finally, selective elimination of FoxO1-negative cells completely inhibited the growth of PDAC cells.

## Materials and Methods

### Specimens from patients

A total of 19 resected PDAC specimens were used in this study. All specimens had been histologically and clinically diagnosed at Department of Medical Oncology, Shanghai First People’s Hospital of Shanghai Jiao Tong University from 2008 to 2013. For the use of these clinical materials for research purposes, prior patient’s consents and approval from the Institutional Research Ethics Committee were obtained. The methods were carried out in accordance with the approved guidelines.

### Cell line culture and treatment

PANC-1 has been generated from a human carcinoma of the exocrine pancreas in 1975^29^, and was purchased from American Type Culture Collection (ATCC, Rockville, MD, USA). PANC-1 cells were maintained in Dulbecco’s modified Eagle’s medium (DMEM, Invitrogen, Carlsbad, CA, USA) supplemented with 15% fetal bovine serum (FBS; Sigma-Aldrich, St Louis, MO, USA) in a humidified chamber with 5% CO_2_ at 37 °C. Diphtheria toxin (DT, Sigma-Aldrich) was freshly prepared and given to the cultured cells at a concentration of 40 nmol/l to specifically eliminate cells expressing diphtheria toxin receptor (DTR).

### Transduction with adeno-associated virus (AAV)

AAV-pFoxO1-GFP-pCAG-luciferase, AAV-pFoxO1-Cre and AAV-pCAG-loxp-DTR-2A-mT-STOP-loxp-mG were prepared as has been previously described^30^,^31^,^32^. mT is membrane tomato red fluorescence and mG is a membrane GFP. This construct has been described previously^33^,^34^. Briefly, we used a pAAV-CAG-GFP plasmid, a pAAV-CAG-luciferase and a pAAV-pCAG-loxp-mT-STOP-loxp-mG plasmid as backbones (all from Clontech, Mountain View, CA, USA), with a packaging plasmid carrying the serotype 8 rep and cap genes and a helper plasmid carrying the adenovirus helper functions (Applied Viromics, LLC. Fremont, CA, USA) to generate AAV in this study. The transgenes (DTR, Cre-2A and a human FoxO1 promoter) were incorporated into the backbone plasmids using proper restriction endonuclease-mediated cloning assay. A full-length human FoxO1 promoter was amplified by PCR with EcoRI-restriction-endonuclease-forward and NheI-restriction-endonuclease-reverse primers, using the human genomic DNA as a template. This construct was ligated with a pCAG-luciferase construct, and then subcloned into the 50-EcoRI and 30-NheI sites of the pAAV-CAG-GFP vector to replace the CAG promoter to generate pAAV-pFoxO1-GFP-pCAG-luciferase. Cre fragment was cut out from a Cre containing plasmid pPC3.1-CMV-Cre with EcoRIII and BamHI restriction endonucleases, and then ligated to the EcoRIII and BamHI sites of plasmid pAAV-FoxO1-GFP-pCAG-luciferase after same enzymes’ cutting to remove GFP-pCAG-luciferase. DTR fragment was cut out from a DTR containing plasmid pFB-CMV-DTR with EcoRIII and BamHI restriction endonucleases, ligated with a 2A, and then incorporated to the EcoRIII and BamHI sites (between loxp and mT) of plasmid pAAV-pCAG-loxp-mT-STOP-loxp-mG after same enzymes’ cutting. The small 2A peptide sequences, when cloned between genes, allow for efficient, stoichiometric production of discrete protein products within a single vector through a novel “cleavage” event within the 2A peptide sequence. Sequencing was performed to confirm the correct orientation of these new plasmids. AAV was prepared by triple transfection of the prepared AAV plasmid, R2C8 (containing AAV2 Rep and AAV8 capsid genes) and plAd5 (containing adenovirus helper genes) into HEK293 cells by Lipofectamine 2000 reagent (Invitrogen). The viruses were purified using CsCl density centrifugation and then titered by a quantitative densitometric dot-blot assay. For cell transduction in vitro, the cells were incubated with AAV at a MOI of 100 for 12 hours.

### Primary Tumor Sphere Culture

Purified tumor cells by flow cytometry were washed, acutely dissociated in oxygenated artificial cerebrospinal fluid and subject to enzymatic dissociation. Tumor cells were then resuspended in tumor sphere media (TSM) consisting of a serum-free DMEM, human recombinant EGF (20 ng/ml; Sigma-Aldrich), bFGF (20 ng/ml; Sigma-Aldrich), leukemia inhibitory factor (10 ng/ml; Sigma-Aldrich) and N-acetylcysteine (60 μg/ml; Sigma-Aldrich), and then plated at a density of 2 × 10^6^ cells/60 mm plate.

### Analysis of CD133, aldefluor, GFP, mT and mG by flow cytometry

CD133 and GFP/mT/mG-based cell analysis and sorting were performed by flow cytometry, based on either PEcy7-conjugated anti-human CD133 antibody (Becton-Dickinson Biosciences, San Jose, CA, USA)-labeling or direct fluorescence. For aldefluor-based cell analysis and sorting, cells were labeled with the Aldefluor Kit (StemCell Technologies, China) according to the manufacturer’s instructions. Flow cytometry was performed using a FACSAria (Becton-Dickinson Biosciences) flow cytometer.

### Cell proliferation assay

For assay of cell growth, cells were seeded into 24 well-plate at 10000 cells per well and subjected to a Cell Proliferation Kit (MTT, Roche, Indianapolis, IN, USA), according to the instruction of the manufacturer. The MTT assay is a colorimetric assay for assessing viable cell number, taking advantage that NADPH-dependent cellular oxidoreductase enzymes in viable cells reduce the tetrazolium dye 3-(4,5-dimethylthiazol-2-yl)-2,5-diphenyltetrazolium bromide (MTT) to its insoluble formazan in purple readily being quantified by absorbance value (OD) at 570 nm. Experiments were performed 5 times.

### Quantitative real-time PCR (RT-qPCR)

MiRNA and total RNA were extracted from cultured cells with miRNeasy mini kit or RNeasy kit (Qiagen, Hilden, Germany), respectively for cDNA synthesis. Quantitative real-time PCR (RT-qPCR) was performed in duplicates with QuantiTect SYBR Green PCR Kit (Qiagen). All primers were purchased from Qiagen. Data were collected and analyzed with the Rotorgene software accompanying the PCR machine, using 2-ΔΔCt method for quantification of the relative mRNA expression levels. Values of genes were first normalized against α-tubulin, and then compared to controls.

### Mouse handling

All mouse experiments were approved by the Institutional Animal Care and Use Committee at Shanghai First People’s Hospital of Shanghai Jiao Tong University (Animal Welfare Assurance). Surgeries were performed in accordance with the Principles of Laboratory Care, supervised by a qualified veterinarian. The methods were carried out in accordance with the approved guidelines. All efforts were made to minimize pain and suffering. Female NOD/SCID mice of 12 weeks of age were used in the current study. Five mice were analyzed in each experimental condition.

### In vivo serial adoptive transfer of tumor cells and imaging by bioluminescence

PANC-1 cells were transduced with AAV-pFoxO1-GFP-pCAG-luciferease, followed by flow cytometry-sorting based on GFP. GFP-positive cells represent FoxO1-positive cells and GFP-negative cells represent FoxO1-null cells. Purified cells (10^2^) were then orthotopically injected into the parenchyma of the pancreas of NOD/SCID mice, as has been described before^35^. The tumor growth after 3 months was monitored and quantified by luminescence levels. Bioluminescence was measured with the IVIS imaging system (Xenogen Corp., Alameda, CA, USA). All of the images were taken 10 minutes after intraperitoneal injection of luciferin (Sigma-Aldrich) of 150 mg/kg body weight, as a 60-second acquisition and 10 of binning. During image acquisition, mice were sedated continuously via inhalation of 3% isoflurane. Image analysis and bioluminescent quantification was performed using Living Image software (Xenogen Corp). The generated tumors were then dissected out and dissociated into single cells. Cells (10^2^) were then orthotopically injected into the parenchyma of the pancreas of new NOD/SCID mice, as a serial adoptive transfer^35^. The tumor growth after another 3 months was monitored and quantified by luminescence levels.

### Statistical analysis

All statistical analyses were carried out using the SPSS 18.0 statistical software package. All data were statistically analyzed using one-way ANOVA with a Bonferoni correction, followed by Fisher’s exact test to compare two groups. For analyses on patients’ specimen, n = 19. All values in cell and animal studies are depicted as mean ± standard deviation from 5 individuals and are considered significant if p < 0.05.

## Results

### CD133-high and aldefluor-high cells expressed high miR-21 and null FoxO1 in PDAC

**Figure 1:**
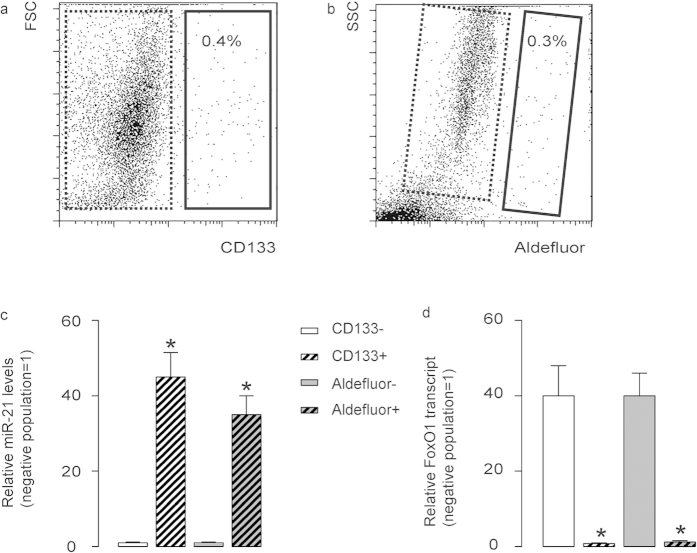
CD133-high and aldefluor-high cells expressed high levels of miR-21 and no FoxO1 in PDAC. (**a,b**) We dissociated resected PDAC specimens from 19 patients and analyzed the tumor cells with CSC marker CD133 (**a**) and aldefluor (**b**) by flow cytometry, shown by representative flow charts. (**c,d**) We analyzed the miR-21 (**c**) and FoxO1 (**d**) transcript levels in CD133-high (solid rectangle in a) vs CD133-low (dotted rectangle in b) populations, and in aldefluor-high (solid rectangle in b) vs aldefluor-low (dotted rectangle in b) populations. ^*^p < 0.05. n = 19. Statistics: one-way ANOVA with a Bonferoni correction, followed by Fisher’s exact test.

Since we have shown a potential role of miR-21-suppressed FoxO1 in the regulation of the growth of PDAC, we thus aimed to see whether the expression of miR-21 and FoxO1 in PDAC cells is uniform or not. We dissociated resected PDAC specimens from 19 patients and analyzed the tumor cells with CSC marker CD133 and aldefluor, the latter of which represents activity of ALDH. We detected CD133 (Fig. 1a) or aldefluor (Fig. 1b) positivity in only a small portion of PDAC cells. These cells were supposed to be CSCs. Then we analyzed the miR-21 and FoxO1 levels in CD133-high vs CD133-low populations, and in aldefluor-high vs aldefluor-low populations (Fig. 1c,d). We found that both ALDH-high and CD133-high cell fractions expressed high miR-21 and nearly null FoxO1, while the negative fractions expressed nearly no miR-21 and high FoxO1 (Fig. 1c,d). These data suggest that the expression of miR-21 and FoxO1 in PDAC from patients is not uniform, and there is a possibility that FoxO1-negative cells could be CSCs in PDAC.

### FoxO1-negative PDAC cells expressed high miR-21, CD133 and aldefluor

**Figure 2:**
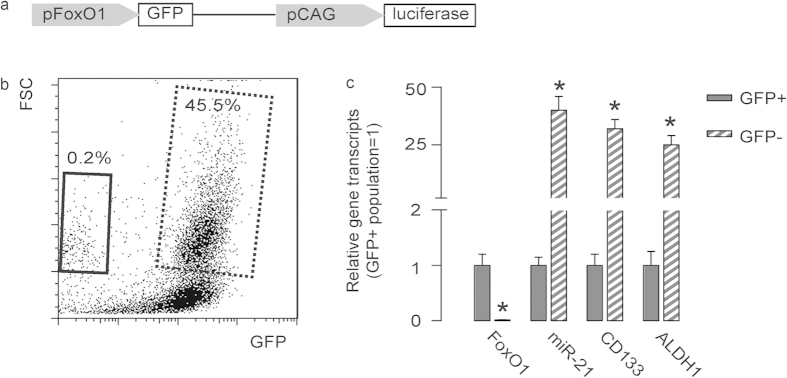
FoxO1-negative PDAC cells expressed high miR-21, CD133 and aldefluor. (**a**) We transduced a human PDAC cell line, PANC-1, with an AVV carrying GFP under the control of a FoxO1 promoter to allow sorting different fractions based on FoxO1 level. This AAV also had a luciferase reporter under the control of a CAG promoter to allow in vivo imaging of the tumor by bioluminescence. (**b**) A representative flow chart is shown for isolation of GFP-positive cells (representing FoxO1-positive cells, dotted rectangle) and GFP-negative cells (representing FoxO1-negative cells, solid rectangle) in the transduced cells. (**c**) Gene transcripts were examined in GFP-negative and GFP-positive populations. ^*^p < 0.05. n = 5. Statistics: one-way ANOVA with a Bonferoni correction, followed by Fisher’s exact test.

In order to prove our hypothesis of FoxO1-negative cells as CSCs in PDAC, we transduced a human PDAC cell line, PANC-1, with an AVV carrying GFP under the control of a FoxO1 promoter to allow sorting different fractions based on FoxO1 transcript level. This AAV also had a luciferase reporter under the control of a CAG promoter to allow in vivo imaging of the tumor by bioluminescence (Fig. 2a). We isolated GFP-positive cells (representing FoxO1-positive cells) and GFP-negative cells (representing FoxO1-negative cells) in the transduced cells (Fig. 2b). First, we confirmed absence of FoxO1 transcripts in the FoxO1/GFP-negative fraction (Fig. 2c). Then, we found that FoxO1/GFP-negative fraction expressed significantly higher levels of miR-21 (about 32 fold higher than FoxO1-positive fraction), CD133 (about 28 fold higher than FoxO1-positive fraction) and ALDH1 (about 24 fold higher than FoxO1-positive fraction) (Fig. 2c). These data suggest that these GFP-negative cells may represent the CSC population in PDAC.

### FoxO1-negative cells grew significantly faster than FoxO1-positive cells

**Figure 3:**
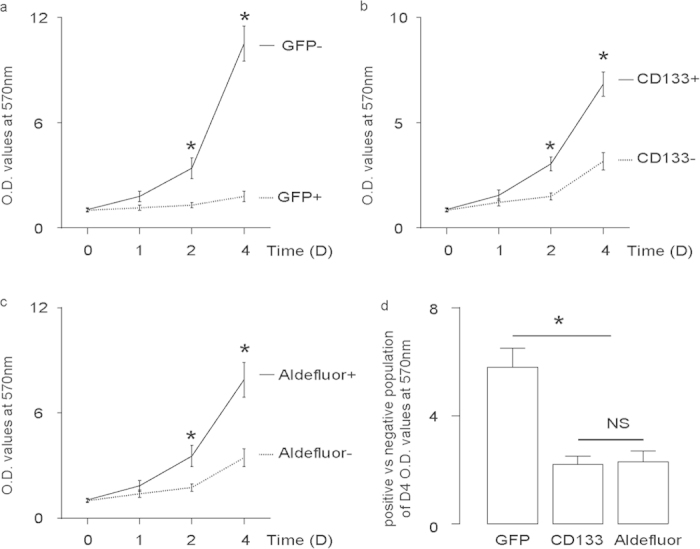
FoxO1-negative cells grew significantly faster than FoxO1-positive cells. (**a,d**) FoxO1-negative cells vs FoxO1-positive cells (**a**), CD133-high vs CD133-low cells (**b**) and aldefluor-high vs aldefluor-low cells (**c**) were used in a MTT assay to examine cell growth. (**d**) The fold increase in growth of FoxO1-negative vs FoxO1-positive cells at D4 is significantly higher than CD133-high vs CD133-low cells, or aldefluor-high vs aldefluor-low cells. ^*^p < 0.05. NS: non-significant. n = 5. Statistics: one-way ANOVA with a Bonferoni correction, followed by Fisher’s exact test.

FoxO1-negative cells and FoxO1-positive cells were used in a MTT assay to examine cell growth. We also isolated CD133-high vs CD133-low cells, and aldefluor-high vs aldefluor-low cells, to compare. We found that FoxO1-negative cells grew significantly faster than FoxO1-positive cells (Fig. 3a), CD133-high cells grew significantly faster than CD133-low cells (Fig. 3b), and aldefluor-high cells grew significantly faster than aldefluor-low cells (Fig. 3c). Of note, the fold increase in growth of FoxO1-negative vs FoxO1-positive cells at D4 is significantly higher than CD133-high vs CD133-low cells, or aldefluor-high vs aldefluor-low cells (Fig. 3d). These data suggest that FoxO1-negative may be a more specific CSC marker in PDAC, than either CD133, or ALDH1/aldefluor.

### CSC property of FoxO1-negative PDACs were supported by tumor sphere formation and tumor formation after adoptive transfer

**Figure 4:**
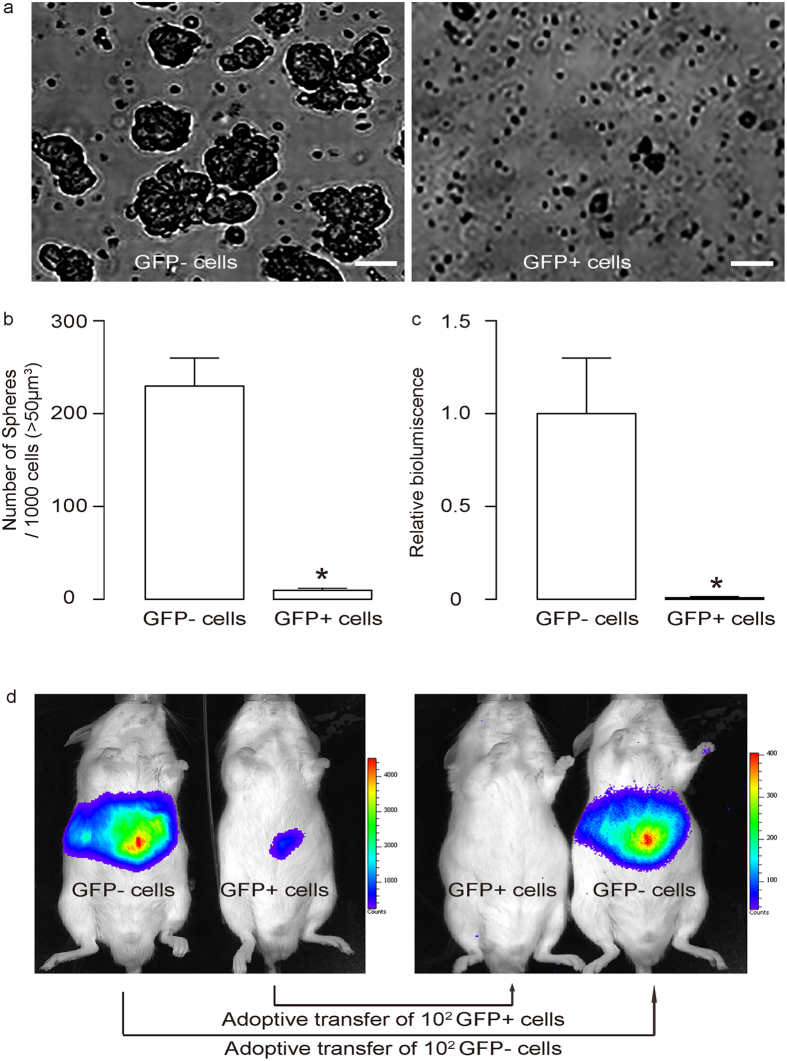
CSC property of FoxO1-negative PDACs were supported by tumor sphere formation and adoptive transfer. (**a,b**) We put FoxO1-negative and FoxO1-positive cells into the tumor sphere media (TSM). We found that only FoxO1-negative cells significantly formed sphere-like structures, while FoxO1-negative cells did not, shown by representative images (**a**), and by quantification (**b**). (**c,d**) Serial adoptive transfer of FoxO1-negative cells, but not serial adoptive transfer of FoxO1-negative cells, developed tumors in receipt NOD/SCID mice, shown by quantification (**c**) and by representative bioluminescent images (**d**). ^*^p < 0.05. n = 5. Statistics: one-way ANOVA with a Bonferoni correction, followed by Fisher’s exact test.

We put FoxO1-negative and FoxO1-positive cells into the tumor sphere media (TSM). We found that only FoxO1/GFP-negative cells significantly formed sphere-like structures, while FoxO1/GFP-positive cells did not (Fig. 4a). Further, serial adoptive transfer of FoxO1/GFP-negative cells, but not serial adoptive transfer of FoxO1/GFP-positive cells, developed tumors in receipt NOD/SCID mice (Fig. 4b). These data support that FoxO1/GFP-negative cells are CSCs in PDAC.

### Elimination of FoxO1-negative cells inhibited cancer growth

**Figure 5:**
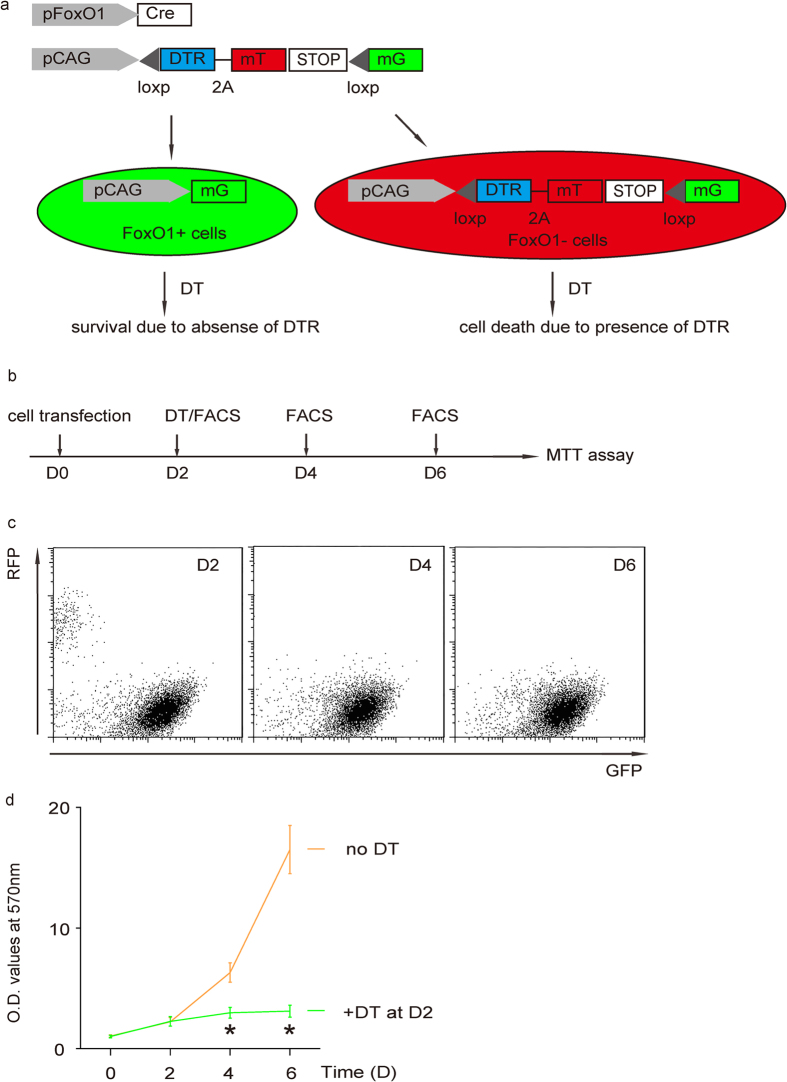
Elimination of FoxO1-negative cells inhibited SCC growth. (**a**) Schematic: We co-transduced a subclone of PANC-1 cells that do not endogenously express DTR with two AAV, AAV-pFoxO1-Cre and AAV-pCAG-loxp-DTR-2A-mT-STOP-loxp-mG. In this model, FoxO1-negative cells express DTR and mT to be red fluorescent and susceptible to DT-induced cell death. FoxO1-positive cells activate Cre recombinase to remove the sequence between two loxps (-DTR-2A-mT-STOP-) and the cells become green fluorescent and lose expression of DTR, and thus become resistant to DT-mediated cell death. (**b**) Schematic: The effect of elimination of FoxO1-negative cells on cell growth in this model was thus examined in a MTT assay 2 and 4 days after DT administration. (**c**) Administration of DT selectively eliminated red fluorescent FoxO1-negative cells, shown by representative flow charts. (**d**) MTT assay. ^*^p < 0.05. n = 5. Statistics: one-way ANOVA with a Bonferoni correction, followed by Fisher’s exact test.

To further confirm that FoxO1/GFP-null cells are CSCs in PDAC, we co-transduced a subclone of PANC-1 cells that do not endogenously express DTR with two AAV, AAV-pFoxO1-Cre and AAV-pCAG-loxp-DTR-2A-mT-STOP-loxp-mG. In this model, FoxO1-negative cells express DTR and mT to be red fluorescent and susceptible to DT-induced cell death. FoxO1-positive cells activate Cre recombinase to remove the sequence between two loxps (-DTR-2A-mT-STOP-) and the cells become green fluorescent and lose expression of DTR, and thus become resistant to DT-mediated cell death^33^,^34^,^36^ (Fig. 5a). The effect of elimination of FoxO1-negative cells on cell growth in this model was thus examined in a MTT assay 2 and 4 days after DT administration (Fig. 5b). We found that after administration of DT, which selectively eliminated red fluorescent FoxO1-negative cells (Fig. 5c), the cells completely stopped growing (Fig. 5d). These data further confirm that FoxO1-negative cells are CSCs in PDAC.

## Discussion

Elucidation of molecular biology underlying PDAC growth is extremely important for improving current therapeutic outcome. Recently, the recognition of CSCs to cancer growth has shed new insight on development of innovative treatments upon PDAC^4^,^5^. Efficiently manipulating CSCs rely largely on the precise characterization of this population among all cancer cells. To date, isolation of CSCs is mainly dependent on cell surface markers, including CD133 and aldefluor^8^,^9^,^10^,^11^,^12^,^16^,^17^,^18^,^19^,^20^,^21^. Although these methods have been shown to substantially enrich the CSCs from various cancers, accumulating evidence has revealed their limitations, and especially being lack of cancer specificity. Thus, identification of CSC markers for a specific cancer is highly needed. Recently, we studied miR-21/FoxO1 interaction in PDAC.

Here, we substantiated our previous work. Based on our findings on the heterogeneous expression of miR-21 and FoxO1 in PDAC from the patient specimen, we hypothesize that FoxO1-negative cells may be CSCs in PDAC. This hypothesis was first supported by comparing the expressions of these factors in purified populations. These studies show that both CD133-high and aldefluor-high cells may be also miR-21-high and FoxO1-negative, suggesting an overlapped expression pattern of these markers. In line with this notion, combined application with CD133, aldefluor, miR-21 and FoxO1 may better characterize CSC population in PDAC.

Further, we compared the power of using these markers to isolate CSCs in PDAC. Since the direct regulation of miR-21 is hard to be studied, we thus used FoxO1-negative as a marker. We found that the increases in cell growth of FoxO1-negative cells vs FoxO1-positive cells were significantly greater than CD133-high vs CD133-low, or aldefluor-high vs aldefluor-low, suggesting a better specificity of FoxO1-negative as a CSC marker than either CD133 or aldefluor/ALDH1 in PDAC cells. Finally, both tumor sphere formation, tumor formation after serial adoptive transfers and studies of selective elimination of FoxO1-negative population all supported that FoxO1-negative cells are CSCs in PDAC. FoxO1 is a protein that is post-translationally regulated, such as phosphorylation, acetylation and ubiquitination and binding protein partners. However, here we show that its transcription control may be also essential for the carcinogenesis of PDACs.

We have also performed limited similar analyses on other PDAC cell lines and on primary PDAC tissue from the patients, we essentially got similar results. Thus, our findings should be not cell-line dependent. The findings in the current study has a clinic-relevance, since treating CSCs may be more effective than targeting the whole tumor mass. Together, FoxO1-negative cells may be a promising therapeutic target of PDAC.

## Additional Information

**How to cite this article**: Song, W. *et al.* FoxO1-negative cells are cancer stem-like cells in pancreatic ductal adenocarcinoma. *Sci. Rep.* **5**, 10081; doi: 10.1038/srep10081 (2015).
